# Prevalence of the Double Burden of Malnutrition among Adolescents: Associations with Lifestyle Behaviors and Clusters of Social Determinants

**DOI:** 10.3390/children11060620

**Published:** 2024-05-22

**Authors:** Raytta Silva Viana, Marcus Vinicius Nascimento-Ferreira, Beatriz D. Schaan, Katia Vergetti Bloch, Kênia Mara Baiocchi de Carvalho, Felipe Vogt Cureau, Augusto César Ferreira De Moraes

**Affiliations:** 1 Postgraduate Program in Public Health, Department of Epidemiology, School of Public Health, University of São Paulo, São Paulo 01246-904, Brazil; raytta@usp.br; 2 YCARE Research Group (Youth/Child Cardiovascular Risk Environmental Research Group), Faculdade de Medicina, University of São Paulo, São Paulo 01246-903, Brazil; 3 Research Group on Health, Physical Activity and Behavior (HEALTHY-BRA), Federal University of Tocantins, Miracema do Tocantins 77650-000, Brazil; 4 Faculty of Medicine, Postgraduate Program in Cardiology and Cardiovascular Sciences, Federal University of Rio Grande do Sul, Porto Alegre 90035-003, Brazil; 5 Division of Endocrinology, Hospital de Clínicas de Porto Alegre, Porto Alegre 90035-903, Brazil; 6 Faculty of Medicine, Postgraduate Program in Endocrinology, Universidade Federal do Rio Grande do Sul, Porto Alegre 90035003, Brazil; 7 Institute of Studies in Public Health, Federal University of Rio de Janeiro, Rio de Janeiro 21941-592, Brazil; 8 Postgraduate Program in Human Nutrition, University of Brasília, Brasília 70910-900, Brazil; 9 The University of Texas Health Science Center at Houston, School of Public Health in Austin, Department of Epidemiology, Michael & Susan Dell Center for Healthy Living, Texas Physical Activity Research Collaborative (Texas PARC) 1836 San Jacinto Blvd., Ste. 510, Austin, TX 78701, USA

**Keywords:** double burden of malnutrition, adolescents, obesity, hypovitaminosis D, prevalence, cluster analysis

## Abstract

The double burden of malnutrition (DBM) is a condition in which malnutrition coexists with overweight, reflecting a new layer of malnutrition. Our objectives were to assess prevalence; test associations between DBM and 24-hour movement behaviors; and investigate whether DBM is associated with clusters of social determinants. Methods: This multicenter cross-sectional study included 1152 adolescents (12 to 17 years old) from four Brazilian cities. Body mass index (BMI, kg/m^2^) was used to estimate overweight, and the adopted cutoff points took into account the curves established for age and sex: Z-score > 1 and ≤2 (overweight) and Z-score > 2 (obesity). The serum concentration of 25-hydroxyvitamin D [25(OH)D] was stratified into three levels: vitamin D deficiency ≤ 20 ng/mL; vitamin D insufficiency = 21–29 ng/mL; optimal vitamin D ≥ 30 ng/mL. We used multilevel Poisson regression models to estimate prevalence ratios (PRs) and their respective 95% confidence intervals (95%CI) and to analyze the association between DBM and covariates. A significance level of *p* < 0.05 was considered. Cluster analyses were performed by applying a combination of hierarchical and non-hierarchical methods. Results: A population prevalence of DBM of 7.3% (95% CI: 5.9–8.9) was revealed. A percentage of 19.2% (95% CI: 17.0–21.6) of adolescents were overweight, and 8.3% (95% CI: 6.8–10.1) were obese. A total of 41.5% (95% CI: 38.7–44.4) had vitamin D deficiency, and 25.8% (95% CI: 23.4–28.4) had vitamin D insufficiency. However, 24-hour movement behaviors were not associated with DBM. Adolescents living in the southern region of the country, from public schools whose mothers have higher education, have a 1.94 [PR = 2.94 (95% CI: 1.20–7.23)] times greater chance of developing DBM. These results highlight the importance of specific factors to improve the nutritional health of adolescents, considering the specific social determinants identified in this study.

## 1. Introduction

Inadequate nutrition, in its various manifestations, is one of the leading causes of illness and death worldwide. Both malnutrition and overweight have long-lasting impacts and increase the likelihood of facing various health problems [1]. The double burden of malnutrition (DBM) refers to the situation where two distinct forms of malnutrition coexist simultaneously. The first form of malnutrition involves the lack of essential nutrients, such as vitamins and minerals, which can lead to either chronic malnutrition (i.e., lack of nutrients over time) or acute malnutrition (when the lack of nutrients is more immediate and severe). The second form of malnutrition is overweight or obesity. DBM represents a contemporary nutritional challenge affecting countries, families, and individuals [2,3].

The “nutrition transition” refers to a change in the dietary patterns of a population over time. In low- and middle-income countries (LMIC), this transition is occurring rapidly [4,5]. There are two main aspects of this transition: micronutrient deficiencies (many people in these countries are not receiving essential nutrients in sufficient quantities to maintain good health); obesity and non-communicable diseases (NCD) (at the same time, there is a significant increase in the prevalence of obesity and NCD). There is growing consensus worldwide that the occurrence of different forms of malnutrition simultaneously is a problem deserving immediate attention [3,4,6]. This recognition is particularly important for certain population groups, such as adolescents and school-age children, who may be at higher risk but have been neglected thus far [7,8,9].

Due to their increasing nutrient and energy requirements for proper growth and development, adolescents are a high-risk group for malnutrition, not only due to their current nutritional status but also with implications for their future development and the risk of developing NCDs such as diabetes mellitus, hypertension, cardiovascular diseases and obesity [10,11,12,13]. Sunlight is essential for the production of vitamin D in the human body, as exposure of the skin to ultraviolet B (UVB) rays leads to the conversion of a precursor of vitamin D into its active form. Consequently, inadequate sunlight exposure can result in insufficient levels of vitamin D in the body [14,15].

Vitamin D plays a crucial role in maintaining bone health, aiding in calcium absorption, and strengthening bones, and it also has an important role in overall body metabolism [16]. When there is not enough vitamin D in the body, especially during the early stages of life, it can cause various health problems over time, as vitamin D has a wide range of health impacts, affecting everything from cognitive function to the risk of developing chronic diseases, cancer, and bone health-related issues [17,18].

Overweight/obesity, in turn, exacerbates the burden of NCD, the development of which is strongly linked to dietary patterns and lifestyle [6,19]. It is predicted that obesity will affect 150 million people aged 10 to 19 worldwide by 2030 [20]. Obesity in children and adolescents is a chronic health condition resulting from a complex interplay of various factors (physiological, psychological, environmental, socioeconomic, and genetic) [21].

Additionally, children and adolescents with obesity face a range of adverse consequences, both in childhood and adulthood, such as cellular dysfunction leading to insulin resistance, changes in insulin production or inefficient insulin utilization resulting in type 2 diabetes, abnormal lipid levels in circulation promoting dyslipidemia, hormonal disorders due to polycystic ovary syndrome, respiratory problems, musculoskeletal complications, and psychological disorders [19,22,23]. An inverse association between circulating 25-hydroxyvitamin D [25(OH)D] and body mass index (BMI) is also observed. This suggests that individuals who are overweight may be more likely to have inadequate levels of vitamin D [24,25].

We recognize the complexity of health-related behaviors and how the time devoted to various activities throughout the day can directly impact adolescent health outcomes. Adhering to all three 24-hour movement guidelines (sleep duration and sedentary behaviors, including screen time) has been associated with benefits in adiposity profile in children and adolescents. However, adolescents, girls, and youth from countries with lower Human Development Index tend not to meet all three 24-hour movement guidelines [26,27].

Previous studies have individually addressed vitamin D deficiency and overweight/obesity [28,29]. The interaction between these conditions, their associations with behavioral factors, and social determinants are still scarce and require investigation [8]. While it is recognized that malnutrition can manifest in different ways, studies on the prevalence of DBM have mainly observed the coexistence of low weight and overweight/obesity at the population level [30,31,32].

However, other nutritional deficiencies are often less explored, such as micronutrient deficiencies (e.g., vitamin D deficiency) and how these conditions coexist at the individual level [30,31,32]. Especially among adolescents in LMIC, where the DBM may pose an even greater challenge due to unfavorable socioeconomic conditions resulting from social disparities that significantly influence health patterns [30,33].

Therefore, the aim of this study is to assess the prevalence of obesity and vitamin D deficiency among adolescents coexisting in the same individual, as well as to investigate how this condition, known as DBM, is associated with 24-hour movement patterns (energy expenditure behaviors) and social determinants.

## 2. Materials and Methods

### 2.1. Study Sample

This study is part of the “Study of Cardiovascular Risk Factors in Adolescents” (ERICA), a multicenter and cross-sectional survey conducted in a school setting. ERICA aimed to estimate the prevalence of metabolic syndrome and other cardiovascular risk factors among adolescents aged 12 to 17 years in Brazilian cities with a population greater than 100,000 inhabitants attending both public and private schools. A detailed description of the sampling design has been provided elsewhere [34].

This study examined a subsample of ERICA, consisting of 1152 adolescents. Participants were fasting for blood collection, as advised by the research team. Adolescents attended morning classes in four Brazilian cities (Fortaleza, Rio de Janeiro, Brasília, and Porto Alegre) located at different latitudes. The cities were chosen based on geographical criteria and the availability of biorepositories during the study. Sample selection for 25(OH)D analysis was conducted via proportional random allocation, ensuring an equitable distribution of sex, age, race, and data collection period, which serves as an indicator of sun exposure. Blood samples were obtained at schools and then transported to research centers in the four participating cities, where they were processed and stored at a temperature of −80 °C [35,36].

### 2.2. Data Collection

In this study, adolescents from randomly selected classes were informed about the research. They were provided with a questionnaire consisting of 115 questions designed to gather information on demographic and lifestyle characteristics. These questions included details about the adolescents’ physical activity, sleep hours, and screen time. The questionnaire was designed to be answered via either an electronic data collector or a personal digital assistant (PDA), LG GM750Q model.

This device was selected due to its portability, ease of use, and ability to accurately and efficiently record responses. During the pre-test and pilot study, repeated measurements were taken to ensure the accuracy of the collected data. Additionally, quality assurance and quality control procedures were adopted, including careful training of the field team and regular monitoring of data collection. The questionnaire employed was partially developed based on instruments used in previous research on risk factors in youth in Brazil. Participants were instructed to answer all questions, and only complete questionnaires were used in the analyses of this study. More information about the PDA, pilot study, and applied questionnaire can be found in a previous study [35].

Data collection took place between February 2013 and November 2014, following the ethical principles of the Helsinki Declaration. The study was approved by the Research Ethics Committee of the Federal University of Rio de Janeiro in January 2009, as well as by the ethics committees of all 26 states and the Federal District. Permission to conduct the study was obtained from all state and local Departments of Education, as well as from all participating schools. Written informed consent was obtained from each student and their legal guardians. During data collection, measures were taken to protect the privacy and confidentiality of the participants. For further details on sampling, study design, and data collection procedures, please refer to previous publications related to the ERICA study [34,35].

### 2.3. Inclusion and Exclusion Criteria

Inclusion criteria—Participants included in the study were adolescents aged 12 to 17 years who were enrolled in selected schools located in Fortaleza, Brasília, Rio de Janeiro, and Porto Alegre. Eligibility was limited to those attending morning sessions who agreed to participate and signed the informed consent form.

Exclusion criteria—Exclusion criteria encompassed students who failed to undergo anthropometric measurements, collect blood, or complete the self-reported questionnaire. Additionally, students with severe or chronic medical conditions that could interfere with participation or affect study outcomes were excluded.

### 2.4. Instruments and Variables

Data from the self-reported questionnaire completed by adolescents were used to examine movement-related habits (sleep duration, screen time, physical activity, and 24-hour movement behavior score), as well as related variables.

#### 2.4.1. Sleep Time

Questions regarding sleep duration were included in the questionnaire developed by the ERICA research team. Participants indicated their usual bedtime and wake-up times during weekdays and weekends. Responses were provided on a predefined scale, with 24 options corresponding to each hour of the day. To calculate the average weekly sleep duration, a formula weighting sleep hours during weekdays and weekends was used as follows: (weekly sleep time × 5 + weekend sleep time × 2)/7, considering adjustments for bedtime and wake-up times [37].

#### 2.4.2. Physical Activity

To measure participants’ level of physical activity, we adapted the Self-Administered Physical Activity Checklist. This instrument contained a list of 24 types of activities, allowing participants to record the frequency (in days) and duration (in hours and minutes) dedicated to moderate to vigorous physical activities (MVPA) over the past 7 days. To calculate the total weekly time spent on MVPA, we multiplied the self-reported frequency and duration for each activity mentioned in the questionnaire. This questionnaire has been previously used in other studies conducted in Brazil, and its version applied in ERICA was validated for use with Brazilian adolescents [38].

#### 2.4.3. Screen Time

Time spent in sedentary screen-based activities was assessed via a single question about the time dedicated to these activities throughout the day: “On a typical weekday, how many hours do you spend watching TV, using the computer, or playing video games?”. Response options ranged in hours from 0 h/day (“I do not engage in these activities”) to ≥7 h/day, with an additional option of “Did not report/Do not remember”. This measure has been previously validated, showing moderate to high accuracy in identifying adolescents who report excessive screen time [39].

#### 2.4.4. The 24-Hour Movement Behaviors

The 24-hour movement behavior is defined as the complete pattern of physical activities and movement-related behaviors performed during a 24-hour period. This encompasses physical activity and sedentary behaviors, such as time spent in front of electronic devices, as well as sleep time [40]. We detail the measurement methods and classification criteria according to the guidelines established for 24-hour movement [37]. We begin with the selection of theoretical definitions for the constructs of interest, such as physical activity, sedentary behavior, and sleep time [34].

We followed the principles of the International Classification of Activities for Time-Use Statistics to code participants’ daily activities over a 24-hour period, starting at midnight and ending at 11:59 PM [41]. This allowed us to operationally define variables for moderate and vigorous physical activity, sedentary behavior (including watching television, playing, using the computer, studying or reading, and passive commuting), and sleep time (both main and incidental). Guidelines recommend at least 60 min of daily MVPA, a maximum of 2 h of screen time per day, and 8 to 10 h of sleep for adolescents aged 13 to 17. Based on these operationalized variables, we employed compositional data analysis to summarize behaviors [42] and further categorized variables into four groups: no behaviors meeting the criteria; one behavior meeting the criteria; two behaviors meeting the criteria; and three behaviors meeting the criteria, following the 24-hour movement guidelines [40].

#### 2.4.5. Covariates

Other analyzed variables included the following: geographical location of cities (Fortaleza, −03°43′; Brasília, −15°46′; Rio de Janeiro, −22°54′; Porto Alegre, −30°01′); sex; age; type of school (public or private). Additionally, the season during data collection was considered (April/May—autumn; June/August—winter; September/October—spring; November/March—summer).

### 2.5. Measurements and Definition of Double Durden of Malnutrition

The DBM is defined at three different levels: population level—when there is a high prevalence of undernutrition and overweight within a given population; household level—in mother–child pairs or within a household, where the mother is overweight, and the child is undernourished; individual level—an individual experiencing both micronutrient deficiency and overweight. In this study, we only considered the individual level of DBM. The assessment of the DBM was conducted considering the simultaneous presence of undernutrition (vitamin D deficiency) and overnutrition (overweight, obesity) in the same individual. To ensure the validity and relevance of our methodologies, our measurements and definitions of DBM were based on the World Health Organization (WHO) guidelines and a comprehensive review of the current scientific literature concerning the potential etiology of selected forms of DBM.

#### 2.5.1. Overweight and Obesity

Overweight and obesity were assessed by measuring weight and height by trained researchers, following standardized procedures. Body weight was recorded using an electronic scale (model P200 M, Leader, Brazil), while height was measured in duplicate with a portable stadiometer (Exact Height, Brazil), with the average of the two measurements taken (with a maximum variation of 0.5 cm between them; if necessary, a third measurement was taken) [35]. From these data, BMI was calculated using the formula BMI = weight (kg)/height (m^2^). Classification of nutritional status was performed according to age- and gender-specific BMI curves from the World Health Organization (WHO). The adopted cutoff points were as follows: Z-score < −3 (very low weight); Z-score ≥ −3 and <−2 (low weight); Z-score ≥ −2 and ≤1 (normal weight); Z-score > 1 and ≤2 (overweight); and Z-score > 2 (obesity) [43].

#### 2.5.2. Serum 25-Hydroxyvitamin D [25(OH)D]

Analysis of 25-hydroxyvitamin D [25(OH)D] was performed on all selected serum samples using a chemiluminescence assay on a LIAISON 5000^®^ analyzer (DiaSorin Inc., Stillwater, MN, USA), with the DiaSorin kit commercially available and acquired via an authorized distributor in Brazil. This assay method has a precision range (coefficient of variation—CV%) of 3% to 8% and an accuracy of 0.92 (95% CI: 0.90–0.94) [44]. All blood collection and storage procedures were standardized, with all 25(OH)D measurements performed at a single central laboratory [36].

Serum 25(OH)D concentration was stratified into three levels: ≤20 ng/mL, 21–29 ng/mL, and ≥30 ng/mL. Following the guidelines of the American Society of Endocrinology, vitamin D deficiency or hypovitaminosis is characterized by 25(OH)D levels below 20 ng/mL, while vitamin D insufficiency is defined by 25(OH)D levels between 21 and 29 ng/mL. A serum level of 25(OH)D of 30 ng/mL or higher was considered ideal [18]. In this study, we used this ordinal classification, assuming that each lower level represents a progressively deficient profile relative to the immediately higher level of 25(OH)D.

### 2.6. Cluster Analyses for Social Determinants

We employed cluster analysis to identify social determinants related to the DBM in adolescents. Three specific social variables were considered: city, type of school (public or private), and maternal education (Incomplete Basic Education; Complete Elementary Education; Complete Secondary Education; Complete Higher Education). We identified five comparable groups, each presenting unique characteristics that allowed us to categorize adolescents into groups with different DBM risk profiles based on specific social determinants.

Cluster G1 is made up of teenagers from the city of Fortaleza, the majority of whom attend private schools and receive predominantly maternal education categorized as Complete Secondary School.

Cluster G2 was determined for adolescents in the city of Porto Alegre, with a higher frequency of public schools, with a predominance of maternal education at the Complete Higher Education level and a higher proportion of DBM.

Cluster G3 included adolescents from the capital of Rio de Janeiro, with a predominance of more than 90% in public schools, with a predominant maternal education of Complete Secondary School and Complete Higher Education combined.

Cluster G4 presents characteristics of four cities (Fortaleza, Rio de Janeiro, Brasília, and Porto Alegre), with a predominance of more than 90% of public schools and a level of maternal education composed exclusively of Complete Elementary Education.

Cluster G5 is formed by adolescents from Brasília, with similar proportions between public and private schools and maternal education level composed with similar proportions for Complete Secondary Education and Complete Higher Education.

### 2.7. Statistical Analyses

For all statistical analyses, we utilized Stata 15 software (Stata Corp., College Station, TX, USA). The analyses were adjusted for the clustered structure of the sample, employing the “svy” command set. We considered the complex sampling method and incorporated all sources of variability from the ERICA sample. Statistical descriptions were presented as means (for continuous variables), percentages (for categorical variables), and 95% confidence intervals (95% CI).

We employed a strategy that combined hierarchical and non-hierarchical analysis to find clusters of similar behavior. Initially, we used the complete linkage method in hierarchical analysis, based on squared Euclidean distances, to measure dissimilarity between clusters. Then, we refined the initial solution via non-hierarchical k-means cluster analysis using the resulting centroids. This process allowed us to group adolescents into clusters with similar behavior patterns regarding DBM [45].

To visualize the hierarchical structure of the clusters, we created a dendrogram that shows how observations are grouped as clusters are combined. This visualization helped us determine the appropriate number of clusters for our study. The identified clusters represent groups of adolescents with similar behavior patterns related to DBM. This allowed us to categorize adolescents into groups with different DBM risk profiles based on specific social determinants.

We adopted multilevel Poisson regression models with random effects intercepts to calculate prevalence ratios (PRs) and their 95% CI. We analyzed the association between the DBM and various covariates, considering two levels of data organization: (i) contextual factors such as city (research center), seasonality, and type of school; and (ii) individual factors, including biological sex, age, and 24-hour behavior score, encompassing physical activity, sedentary behavior (screen time), and sleep time [46]. We considered a 10% modification in the β coefficient of any variable already present in the model as significant. Variables that showed *p* < 0.20 in univariate analysis were included in multivariate multilevel models. The adopted significance level was *p* < 0.05.

## 3. Results

We assessed 1152 Brazilian adolescents from four capital cities, and their characteristics are presented in Table 1. We found a population prevalence of more than 7% for DBM among adolescents, with higher rates among girls and individuals from public schools. More than two-thirds of the adolescents had low levels of vitamin D, and 27.5% were overweight (overweight and obesity). Over half of the sample did not engage in 60 min or more of moderate to vigorous-intensity physical activity per day, did not have adequate sleep time (8–10 h per night), and spent more than 2 h per day in front of screens.

Table 2 displays the prevalence of DBM according to independent variables. Among the research centers, Porto Alegre significantly exhibits the highest prevalence compared to the other cities. No significant differences in the prevalence of the double burden of malnutrition were found when analyzing other independent variables.

The multilevel analysis is presented in Table 3. The prevalence ratio (PR) of DBM in Porto Alegre was significant, showing a PR of 3.93 (95% CI: 1.88–8.21) times higher for Porto Alegre compared to the city of Fortaleza. All seasonality PRs presented significant values for the reference variable “Summer”. Each cluster is numbered and named G1, G2, G3, G4, or G5. We identified five comparable clusters based on the variable’s city, type of school, and maternal education. The Dendrogram with the distribution of identified clusters is available in Figure S1 of the Supplementary Materials. Cluster Group 2, which only included data from Porto Alegre from public schools with a high level of maternal education, was the only one with statistically significant values (*p* < 0.05).

## 4. Discussion

Our objective was to assess the prevalence of DBM (overweight and vitamin D deficiency) at the individual level among adolescents and to test the association of DBM with 24-hour energy expenditure behaviors (sleep time, physical activity, sedentary time) and social determinants (maternal education, type of school, city of residence). Our results demonstrate that the prevalence of DBM is alarming in this population and is more strongly associated with female adolescents and those who attend public schools. Furthermore, regarding social determinants, a higher likelihood of presenting DBM was found among the cluster of adolescents from lower latitude areas, attending public schools, and whose mothers have higher education. Sleep duration, screen time, and levels of physical activity were not associated with DBM.

According to the standards established by the WHO for nutritional health indicators, the prevalence found in our study represents a warning sign [47], indicating the simultaneous presence of health issues related to vitamin D deficiency and overweight. The coexistence of these conditions has assumed the status of a double burden in a significant portion of the adolescent population, suggesting additional complexity in the health landscape [48]. Furthermore, the mutual negative effects between vitamin D deficiency and excess fat not only have isolated impacts on health but also negatively influence each other via complex metabolic interactions [24].

The higher prevalence of DBM among girls emerges as a complex and significant finding, indicating the influence of biological, behavioral, and environmental aspects. Considering the biological and hormonal differences between genders, issues related to sexual maturation and hormonal development may play a role in the observed disparity. This disparity may be attributed to biological variations in energy demands and body composition between males and females, related to growth rate and timing of sexual maturation [49,50,51].

Additionally, cultural factors, social aspects, and gender norms can impact choices and opportunities related to specific dietary behaviors and patterns of physical activity in female adolescents, influencing both overweight and vitamin D deficiency. Men tend to be more physically active than women [52] and due to cultural influences, girls generally spend more time at home than boys, resulting in physical inactivity, less sun exposure, and contributing to increased overweight [53].

Adolescents from Porto Alegre (latitude: −30°01′), characterized by a lower latitude, demonstrated a higher susceptibility to DBM compared to those residing in Fortaleza (latitude: −03°43′). This could be explained by the variation in latitude between these cities contributing to differences in seasonality, intensity of available ultraviolet B (UVB) radiation, and environmental conditions (climate/weather) [54,55]. Also, individual factors such as dietary intake of vitamin D, intensive use of sunscreen, personal time habits, and pattern of sun exposure, especially during specific seasons, along with exposed skin area and skin pigmentation [56,57,58].

Promoting health in regions with lower latitude involves encouraging outdoor physical activities, proven to be effective in raising serum vitamin D levels [59,60]. Furthermore, it is crucial to recognize the geographical and cultural diversity within the country, adapting policies to local realities, considering factors such as cultural practices and lifestyle habits.

Distinct clusters, categorized by specific social variables, highlight the significant influence of factors such as city, type of school, and maternal education on the prevalence of DBM. Cluster G2, composed of adolescents from Porto Alegre, mainly from public schools, whose mothers have the highest levels of education, showed the highest prevalence of DBM, indicating an association with specific social, economic, and cultural characteristics of this region.

Our findings contrast with the protective association often observed between maternal education and nutrition in Latin American countries. However, the relationship between maternal education and nutritional health cannot be understood in isolation [61,62]. According to The State of Food Security and Nutrition in the World (SOFI) report, the primary and underlying reasons for undernutrition are related to environmental, economic, and sociopolitical factors, with poverty being a fundamental factor in this context [63].

In a recent cohort study involving a sample of 2782 mothers and their children, the association between consumption patterns and socioeconomic indicators was investigated, revealing that within the group of mothers with moderate to high education levels, low family finances were an additional risk factor for unhealthy dietary patterns [64]. The research indicates that families with low incomes often face financial constraints that limit their ability to purchase a variety of nutritious foods [65]. This situation ultimately results in considerable differences in food consumption among children from families with different economic conditions [66].

Maternal education can influence both children’s dietary habits and sedentary behaviors. Studies indicate that mothers with a high level of education and who work outside the home commonly face time constraints and long hours of maternal work, or unemployed parents are associated with prolonged screen time in the pediatric population [67,68]. The association between maternal education and the level of vitamin D in adolescents, as well as the exact factors mediating this association, require further investigation [69]. Maternal education, despite its importance, may represent a barrier to outdoor activities in a specific context, such as Porto Alegre, where mothers have higher levels of education but are also exposed to an environment with higher levels of inequality and per capita income [67,70].

Regarding the predominance of public schools in group G2, it is important to highlight notable socioeconomic disparities between students attending public and private schools. Research indicates variations in the prevalence of overweightness according to the socioeconomic characteristics of the population, suggesting an inverse relationship between body mass index and socioeconomic status [71,72,73]. Furthermore, it is crucial to consider the availability of outdoor physical activity spaces and the infrastructure of public schools. In many regions, especially urban areas, public schools face challenges in providing adequate facilities for physical activities, such as sports fields or outdoor recreation areas. The lack of access to these spaces may limit opportunities for regular physical exercise and contribute to deficiencies in vitamin D and other nutritional imbalances, such as overweightness [71,74,75].

This study provides greater insight into specific factors that can improve the nutritional health of adolescents; however, the limitations and strengths of the research methodology need to be taken into consideration. Among the limitations, data collection in schools stands out, introducing a potential selection bias by not capturing adolescents outside the school environment. Relying on self-report for behaviors such as physical activity, screen time, and dietary intake may be subject to inaccuracies and memory biases. The disparity in the number of female participants compared to male participants. Although we observed a stronger association between the double burden of malnutrition (DBM) prevalence among female adolescents, it is important to acknowledge that the sample of females was significantly larger than that of males (703 females versus 409 males). This gender disparity may have influenced our analyses and interpretations of the results. However, we suggest that additional analyses considering these gender differences be explored in future studies for a more comprehensive understanding of the determinants of DBM in adolescents. Despite the limitations of BMI as a metabolic health indicator, it is crucial to note that obesity measurement in this study was solely conducted using BMI, without incorporating other body composition measurement methods such as anthropometry with perimeters or skinfolds, bioimpedance, or Dual-Energy X-ray Absorptiometry (DEXA) [76]. This approach may compromise the accuracy of obesity assessment and limit a comprehensive understanding of body fat distribution among participants. Therefore, future research in this area should consider incorporating a variety of body composition measurement methods to enhance the precision and comprehensiveness of obesity assessments and their health implications. Such an approach can broaden the understanding of obesity’s multifaceted nature and its associations with various health risk factors. The lack of detailed information on certain behaviors, such as sleep quality and specific dietary characteristics, limits the comprehensive understanding of these factors. However, the study’s strengths include the sample size, covering diverse geographical regions and socioeconomic groups. Cluster analysis allowed the identification of distinct patterns of behavior related to the DBM, providing relevant insights for intervention strategies and our study serves as a foundation for national-level policies and recommendations. The adoption of rigorous criteria for assessing vitamin D deficiency and excess weight enhances the reliability of the results. Although the study did not find expected associations, the need for further research is evident to better understand the relationship between 24-hour Movement Behaviors, the other variables studied, and DBM, exploring more specific data and investigating additional mediating factors.

## 5. Conclusions

In conclusion, the evidence presented in this article reveals that the prevalence of DBM is a cause for concern among Brazilian adolescents. Particularly, the prevalence of DBM was higher in girls and public schools. Contrary to expectations, sleep duration, screen time, and physical activity levels were not associated with DBM. Still, adolescents from lower latitude areas who attend public schools and whose mothers have higher education are more likely to have DBM. Although additional studies are needed, we can conclude that the components that contribute to DBM appear to be complex and multifaceted, driven by a combination of factors such as gender, type of school, region of residence, and maternal education.

## Figures and Tables

**Table 1:** Characteristics and prevalence of double burden of malnutrition among Brazilian adolescents: ERICA Study, Brazil, 2014.

Independent Variables	Total *n* = 1152	Prevalence (%)	(95% CI)
Research center			
Fortaleza	314	27.2	24.7–29.9
Rio de Janeiro	279	24.2	21.8–26.7
Brasília	268	23.2	20.9–25.7
Porto Alegre	291	25.2	22.8–27.8
School type			
Public	832	72.2	69.5–74.7
Private	320	27.7	25.2–30.4
Seasonality			
Summer	14	1.2	0.7–2.0
Spring	289	25.0	22.6–27.6
Autumn	316	27.4	24.9–30.0
Winter	533	46.2	43.3–49.1
Biological Sex			
Female	703	61.0	58.1–63.8
Male	449	38.9	36.1–41.8
Age			
Mean	1.152	14.8	14.7–14.9
Maternal Education			
Incomplete Basic Education	65	6.9	5.4–8.7
Complete Elementary Education	313	33.5	30.5–36.6
Complete High School	301	32.2	29.3–35.3
Complete Higher Education	254	27.2	24.4–30.1
Screen time			
>2 h/day	677	62.6	59.7–65.5
≤2 h/day	403	37.3	34.4–40.2
Sleep time			
<8 or >10 h/night	693	65.5	62.6–68.3
8–10 h/night	364	34.4	31.6–37.3
Moderate to vigorous physical activity			
<60 min/day	618	57.0	54.0–59.9
≥60 min/day	466	42.9	40.0–45.9
24-hour Movement Behavior Score ^a^			
0 behavior	233	24.9	22.2–27.8
1 behavior	397	42.5	39.4–45.7
2 behaviors	241	25.8	23.1–28.7
3 behaviors	62	6.6	5.2–8.4
Nutritional status			
Thinness	19	1.6	1.0–2.5
Normal weight	811	70.7	68.0–73.2
Overweight	221	19.2	17.0–21.6
Obesity	96	8.3	6.8–10.1
25(OH) D levels ^b^			
≥30 ng/mL	375	32.5	29.9–35.3
21–29 ng/mL	479	41.5	38.7–44.4
≤20 ng/mL	298	25.8	23.4–28.4
Double burden of malnutrition ^c^			
No	1.068	92.7	91.0–94.0
Yes	84	7.3	5.9–8.9

^a^ 24-hour movement behavior score measured 24-hour behaviors in physical activity, sedentary behavior, and screen time; ^b^ 25(OH)D = vitamin D; ^c^ Double burden of malnutrition = coexistence of overnutrition plus micronutrient deficiencies.

**Table 2:** Prevalence (%) of double burden of malnutrition according to the independent variables studied among Brazilian adolescents in the ERICA Study, Brazil 2014.

Independent Variables	Double Burden of Malnutrition (Overweight/Obesity Plus 25(OH)D Lower than 21–29 ng/mL)	
	%	(95% CI)
Research center		
Fortaleza	5.4	2.9–9.9
Rio de Janeiro	3.6	2.0–6.4
Brasília	3.7	2.1–6.6
Porto Alegre	16.9 *	12.1–23.1
School type		
Public	5.3	3.9–7.3
Private	6.0	3.6–9.7
Seasonality		
Summer	0	--
Spring	4.1	2.2–7.6
Autumn	4.8	2.6–8.8
Winter	7.3	5.4–9.8
Biological sex		
Female	5.5	3.9–7.6
Male	5.5	3.7–8.0
Screen time		
>2 h/day	5.9	4.2–8.4
≤2 h/day	5.1	3.1–8.0
Sleep time		
<8 or >10 h/night	5.1	3.6–7.1
8–10 h/night	5.0	3.2–7.7
Moderate to vigorous physical activity		
<60 min/day	4.3	3.0–6.1
≥60 min/day	7.0	4.9–9.8
24-hour movement Behavior Score ^a^		
0 behavior	2.6	1.4–4.6
1 behavior	6.3	4.0–9.8
2 behaviors	5.4	3.3–8.6
3 behaviors	2.4	0.5–9.8

^a^ 24-hour movement behavior score measured 24-hour behaviors in physical activity, sedentary behavior, and screen time. Significant differences (*p* < 0.05) are highlighted (*) 95% CI = 95% confidence intervals.

**Table 3:** Prevalence ratio of double burden of malnutrition according to individual and contextual variables among Brazilian adolescents in the ERICA Study, Brazil, 2014.

Variables	Double Burden of Malnutrition (Overweight/Obesity Plus 25(OH)D Lower than 21–29 ng/mL)	
	PR	(95% CI)
Research center		
Fortaleza ^c^	-	-
Rio de Janeiro	0.94	0.33–2.64
Brasília	0.79	0.20–3.00
Porto Alegre	**3.93**	**1.88–8.21**
Seasonality		
Summer ^c^	-	-
Spring	**6.15**	**1.92–1.97**
Autumn	**3.25**	**9.04–1.17**
Winter	**9.16**	**3.34–2.51**
Biological sex		
Female ^c^	-	-
Male	0.67	0.38–1.16
Age (years)	1.01	0.72–1.04
12 ^c^	-	-
13	0.84	0.29–2.43
14	0.79	0.26–2.43
15	0.99	0.37–2.62
16	0.84	0.32–2.17
17	1.04	0.41–2. 65
Type of school		
Public ^c^	-	-
Private	1.04	0.56–1.93
Cluster ^a^		
Group 1 *^c^	-	-
Group 2 **	**2.94**	**1.20–7.23**
Group 3 ***	0.99	0.35–2.82
Group 4 ****	0.83	0.26–2.57
Group 5 *****	0.96	0.35–2.59
Maternal education		
Incomplete Basic Education ^c^	-	-
Completed elementary school	0.40	0.12–1.26
Completed high school	0.62	0.14–2.61
Completed college degree	0.47	0.12–1.84
Screen time		
>2 h/day ^c^	-	-
≤2 h/day	0.79	0.35–1.77
Sleep time		
<8 or >10 h/night ^c^	-	-
8–10 h/night	1.16	0.58–2.33
Moderate to vigorous physical activity		
<60 min/day ^c^	-	-
≥60 min/day	1.06	0.57–1.95
24-hour movement Behavior Score ^b^		
0 behavior	2.16	0.20–17.37
1 behavior	4.93	0.66–36.42
2 behaviors	3.99	0.53–30.08
3 behaviors ^c^	-	-

^a^ Clusters (variables city, maternal education, and type of school) are numbered, named, and with their respective prevalence (* is considered as a reference and represents data associated with the city of Fortaleza; ** represents the data set associated with the city of Porto Alegre; *** refers to data related to the city of Rio de Janeiro; **** covers information from all four cities (Fortaleza, Porto Alegre, Rio de Janeiro and Brasília); ***** is linked to the city of Brasília) ^b^ 24-hour movement behavior score measured 24-hour behaviors in physical activity, sedentary behavior, and screen time. e = exponential value. Significant differences (*p* < 0.05) are highlighted ^c^ considered as the reference group ^d^ Significant associations are in bold *p* < 0.05; 95% CI = 95% confidence intervals.

## Data Availability

Data are contained in the article.

## Supplementary Materials

The following supporting information can be downloaded at: https://www.mdpi.com/article/10.3390/children11060620/s1, Figure S1: Dendrogram with the Distribution of Identified Clusters.
