# Supplementary figures and images for: Prevalence of the Double Burden of Malnutrition among Adolescents: Associations with Lifestyle Behaviors and Clusters of Social Determinants
Source: Children (Basel). 2024 May 22;11(6):620. doi: 10.3390/children11060620 (PMC11201642; doi:10.3390/children11060620)

**Figure S1:** Dendrogram with the Distribution of Identified Clusters

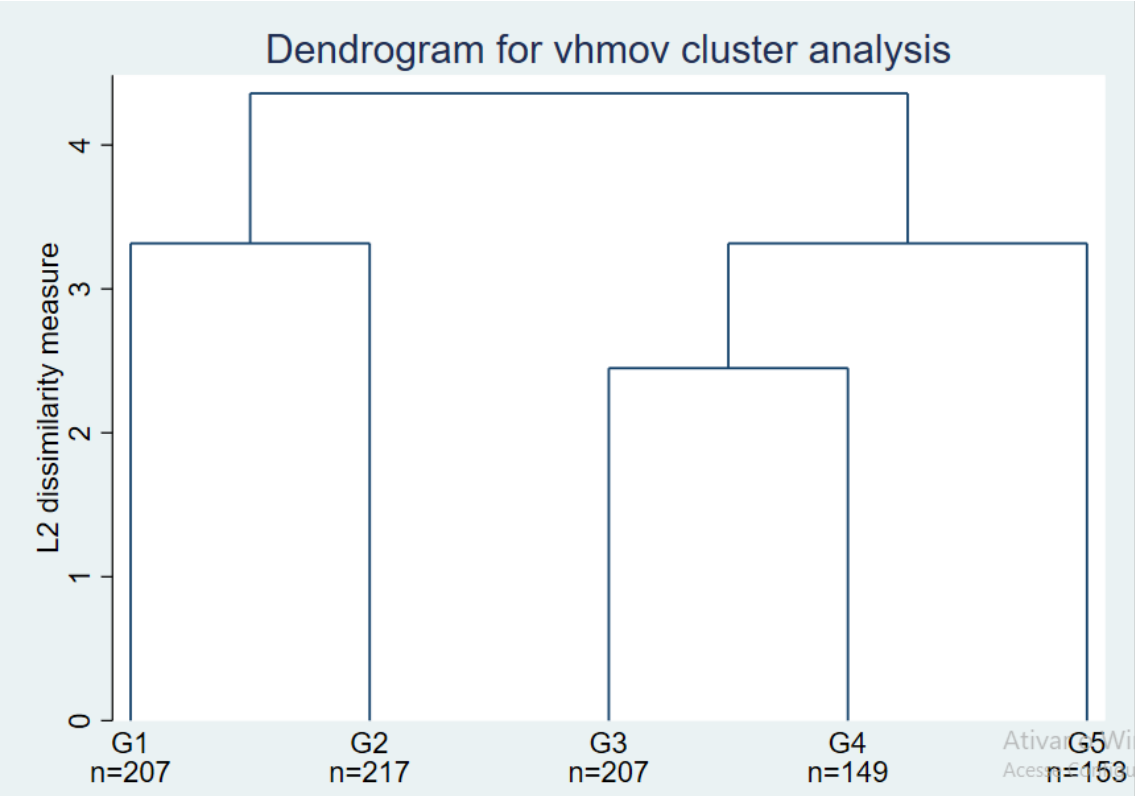

Supplement: Supplementary file 1 [file children-11-00620-s001.zip › children-3001625-supplementary.pdf]
